# A Medical Imbroglio

**Published:** 1875-07

**Authors:** 


*A Medical Imbroglio.*—There was an exciting occurrence at a meeting of the Richmond, Virginia, Academy of Medicine, held June 17th, which resulted in the arrest of several prominent physicians of that city, who were released on bail of $1,000 for a further appearance. On Friday a hearing was had before a Justice of the Peace, when all were discharged except Drs. Hunter, McGuire and J. S. Welford, who were placed under bond of $2,500 each to keep the peace towards each other for twelve months. These gentlemen are amongst the most eminent medical practitioners in Richmond, and the origin of the difficulty between them is thus related: Dr. McGuire was called on about two weeks since to see a lady whose symptoms he found to be very alarming. The usual and simplest medicines in such cases were resorted to, but those failing, and the condition of the patient growing worse, he gave the lady hypodermically five minims of Magendie's solution of morphia. In twenty minutes afterward she expressed herself releived from pain, and the physician left. At twelve o'clock P. M. Dr. McGuire was again sent for, the lady's condition growing evidently and seriously worse, but he being absent from home, the nearest physicians, Drs. Welford and Magill, were summoned. The lady had had a convulsion. Dr. Magill administered an enema of assafœtida and aromatic spirits of ammonia. Soon after convulsion, more violent than the first. Dr. J. S. Welford then arrived, and at his instance another ounce of asafœtida was repeated and an injection of a solution of caffein was made as soon as it could be obtained. A solution of atropia was then sent for. but before it arrived the lady had died. A little later Dr. McGuire arrived, and finding his patient dead, inquired: "Gentlemen, what is your opinion as to the cause of the lady's death?" to which Dr. Magill, after hesitation, responded, "Doctor, if you insist upon a reply, we think she died from opium poisoning." This was regarded by Dr. McGuire as a thrust at his professional skill and reputation, and as soon as the Academy of Medicine met he reported the matter, and asked an expression of opinion as to his treatment of the case, receiving a strong indorsement from a number of gentlemen who spoke upon the subject, and who cited the best medical authorities approving the course pursued. Dr. Mc.Guire afterwards learning that Dr. Welford had made repeated and unprofessional remarks about him, preferred charges, which were referred to the Committee on Ethics. who exonerated Dr. Welford of the charges of unprofessional conduct. Dr. McGuire, at a meeting of the Academy on Thursday night, rising to a personal explanation, stated that he had been met upon the streets and in the houses of his patients by slanders circulated concerning him by Dr. Welford, and he demanded of that gentleman a retraction or a denial that he had spread such reports. Dr. Welford sat on the opposite side of a large table, around which were seated the assembled M. D's. of Richmond, with a pen knife in his hand, paring his finger nails. He did not reply to Dr. McGuire, who thereupon stigmatized him as "a liar and a trickster." As soon as these words were uttered the entire assemblage sprang to their feet, Dr. Welford, with the rapidity of thought, hurling his open knife at Dr. McGuire. The missile passed harmlessly by McGuire but wounded Dr. Harris, who was behind him, in the wrist. A scene of great excitement ensued, which was ended by the arrest of the parties, as related above. Nobody considers the affair ended, and a duel is believed to be inevitable, notwithstanding the principals have been placed under heavy bonds to keep the peace. The Academy of Medicine having appointed a committee to investigate the charges preferred against Drs. Welford and Magill by Dr. Mc Guire, alleging malpractice by which a lady's death was induced the committee report that "after a careful examination and discussion of the evidence submitted to them your committee respectfully report that they find Drs. Welford and Magill not guilty of any breach of medical ethics in relation to the case reported by Dr. McGuire, May 6th, 1875." The committee consisted of F. D. Cunningham, chairman; John G. Skelton, C. Henry Perrow, O. A. Crenshaw, and M. M. Walker. The report of the committee was received and unanimously adopted by the Academy of Medicine.

